# Algorithm for Detection and Quantification of Hyperreflective Dots on Optical Coherence Tomography in Diabetic Macular Edema

**DOI:** 10.3389/fmed.2021.688986

**Published:** 2021-08-18

**Authors:** Haifan Huang, Liangjiu Zhu, Weifang Zhu, Tian Lin, Leonoor Inge Los, Chenpu Yao, Xinjian Chen, Haoyu Chen

**Affiliations:** ^1^Joint Shantou International Eye Center, Shantou University and the Chinese University of Hong Kong, Shantou, China; ^2^Department of Ophthalmology, University Medical Center Groningen, University of Groningen, Groningen, Netherlands; ^3^School of Electronics and Information Engineering, Soochow University, Suzhou, China

**Keywords:** diabetic macular edema, diabetic retinopathy, optical coherence tomography, deep learning algorithm, hyperreflective dots

## Abstract

**Purpose:** To develop an algorithm to detect and quantify hyperreflective dots (HRDs) on optical coherence tomography (OCT) in patients with diabetic macular edema (DME).

**Materials and Methods:** Twenty OCTs (each OCT contains 128 b scans) from 20 patients diagnosed with DME were included in this study. Two types of HRDs, hard exudates and small HRDs (hypothesized to be activated microglia), were identified and labeled independently by two raters. An algorithm using deep learning technology was developed based on input (in total 2,560 OCT b scans) of manual labeling and differentiation of HRDs from rater 1. 4-fold cross-validation was used to train and validate the algorithm. Dice coefficient, intraclass coefficient (ICC), correlation coefficient, and Bland–Altman plot were used to evaluate agreement of the output parameters between two methods (either between two raters or between one rater and proposed algorithm).

**Results:** The Dice coefficients of total HRDs, hard exudates, and small HRDs area of the algorithm were 0.70 ± 0.10, 0.72 ± 0.11, and 0.46 ± 0.06, respectively. The correlations between rater 1 and proposed algorithm (range: 0.95–0.99, all *p* < 0.001) were stronger than the correlations between the two raters (range: 0.84–0.96, all *p* < 0.001) for all parameters. The ICCs were higher for all the parameters between rater 1 and proposed algorithm (range: 0.972–0.997) than those between the two raters (range: 0.860–0.953).

**Conclusions:** Our proposed algorithm is a good tool to detect and quantify HRDs and can provide objective and repeatable information of OCT for DME patients in clinical practice and studies.

## Introduction

The global prevalence of diabetes was 415 million in 2015, and this number is estimated to increase to 642 million by 2040 (1). About one third of diabetic patients will develop diabetic retinopathy, which is the major cause of blindness in the working-age population in the world (2, 3). Diabetic macular edema (DME) is one of the primary causes of vision loss in patients with diabetic retinopathy (4).

DME is characterized by an excessive accumulation of intraretinal or subretinal fluid in the macula and can best be detected by optical coherence tomography (OCT) (4). High-resolution OCT images give information on the severity of DME (4) and on accompanying morphological changes, such as intraretinal cysts, subretinal fluid, disorganization of retinal inner layers, and hard exudates (5, 6). Hard exudates are lipid deposits that can be seen on fundus photographs and give hyperreflective signals on OCT (7). Recently, small hyperreflective dots (HRDs) unseen on fundus photographs but visible on OCT have been reported in diabetic eyes and were hypothesized to be activated microglia (5, 6, 8). A recent study indicated that these small HRDs can best be distinguished from hard exudates by their diameter, reflectivity, and back shadowing status (8). Both hard exudates and activated microglia are probably involved in the pathogenesis of diabetic retinopathy development but playing different roles, and are related to treatment responsiveness in DME patients (9–12). However, current clinical studies investigated HRDs with manual calculation on selected OCT sections (10, 13, 14), which may be time consuming and induce subjective bias for analysis.

Artificial intelligence is a branch of computer science that aims to perform tasks by simulating intelligent human behavior (15, 16). Machine learning is a subfield of artificial intelligence that uses statistical techniques to enable computers to learn on their own without being explicitly programmed (17). Deep learning is the newest component of machine learning and is widely adopted in image recognition in ophthalmology (15). Methods for automatic quantification of HRDs on OCT using artificial intelligence were published recently (18–20). In these papers, HRDs were selected without consideration of differentiation between hard exudates and smaller hyperreflective dots hypothesized to be activated microglia.

Thus, we plan to develop a deep learning algorithm to quantify HRDs on OCT in DME patients and differentiate them into hard exudates and small HRDs, and to investigate the consistency of automatic and manual calculation of HRDs on OCT.

## Materials and Methods

### Data Collection

Twenty OCTs from 20 DME patients diagnosed at Joint Shantou International Eye Center (JSIEC) of Shantou University and the Chinese University of Hong Kong were included in this study. DME was defined as average central retinal thickness >300 μm within the 6-mm ETDRS circle on the OCT report (21, 22) and without other retinal diseases that may cause macular edema. OCT files with poor quality evaluated by Topcon OCT built-in software with a score of TopQ Image Quality <30 were excluded. All patients underwent examination with the Topcon 3D OCT-2000 device (Topcon, Tokyo, Japan) using the macula mode. The OCT scanning area was 6 × 6 × 2 mm^3^ centered on the fovea, corresponding to 128 × 512 × 885 pixels. This study adhered to the tenets of Declaration of Helsinki and was approved by the Institutional Review Board of JSIEC of Shantou University and the Chinese University of Hong Kong (No. 19-006). Informed consents are waived due to the retrospective nature of the study.

### Algorithm Development

Famicom Disk System data were exported anonymously from the Topcon 3D OCT-2000 device and transformed to ITK MetaImage Header files.

**Figure 1:**
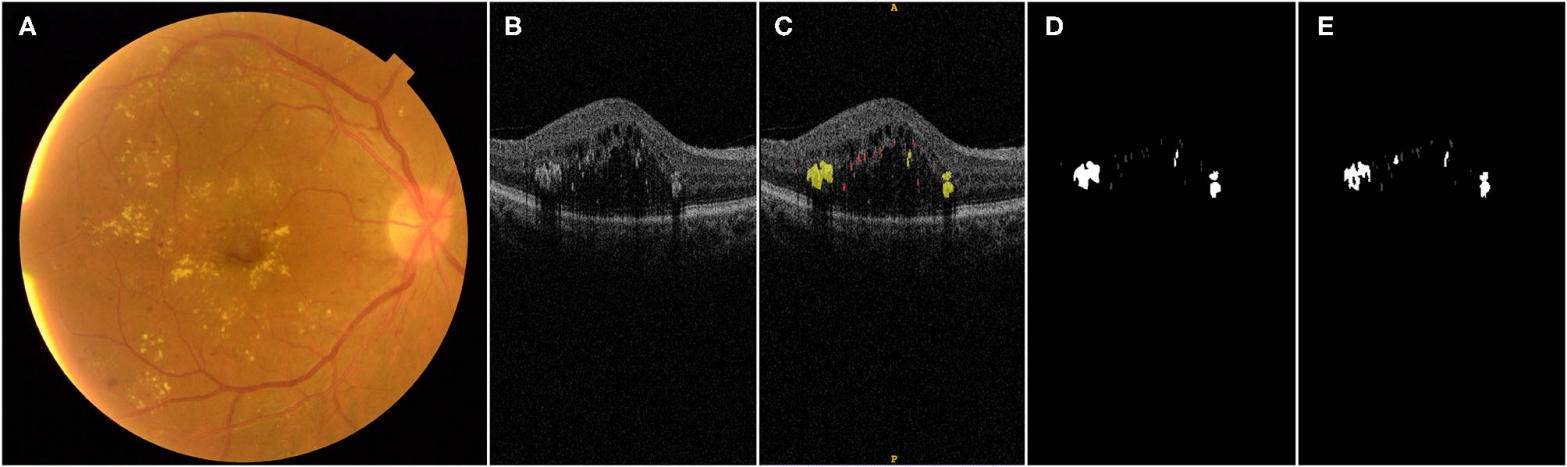
Illustration of manual labeling of HRDs on OCT b scans in DME patients: **(A)** fundus photograph of a patient with DME; **(B)** one OCT b scan of the same eye with different HRDs; **(C)** manual label of HRDs of the OCT b scan shown in **(B)**, hard exudates with diameter larger than 40 μm, presence of back shadowing and reflectivity similar to RPE–Brush complex are labeled with yellow color; small HRDs with diameter between 20 and 40 μm and similar reflectivity to nerve fiber layer without back shadowing are labeled with red color; **(D)** output of manual label by rater 1; **(E)** output of label by proposed algorithm.

Manual labeling of HRDs on 2560 OCT b scans (20 OCTs, each containing 128 b scans) was performed by two ophthalmologists (rater 1: H.H. and rater 2: T.L.) using itk-SNAP software (23). Two types of HRDs, hard exudates and small HRDs, were identified and labeled separately with different colors (Figure 1). Hard exudates were defined as particles larger than 40 μm with back shadowing and reflectivity similar to the retinal pigment epithelium–Bruch complex (8). Small HRDs were defined as particles 20 to 40 μm in diameter (10, 24) with similar reflectivity to the nerve fiber layer without back shadowing on OCT within the neurosensory retina (8). Signals smaller than 20 μm were regarded as noise and excluded.

The algorithm flow mainly includes two steps: automatic segmentation of HRDs and classification of the segmentation results.

**Figure 2:**
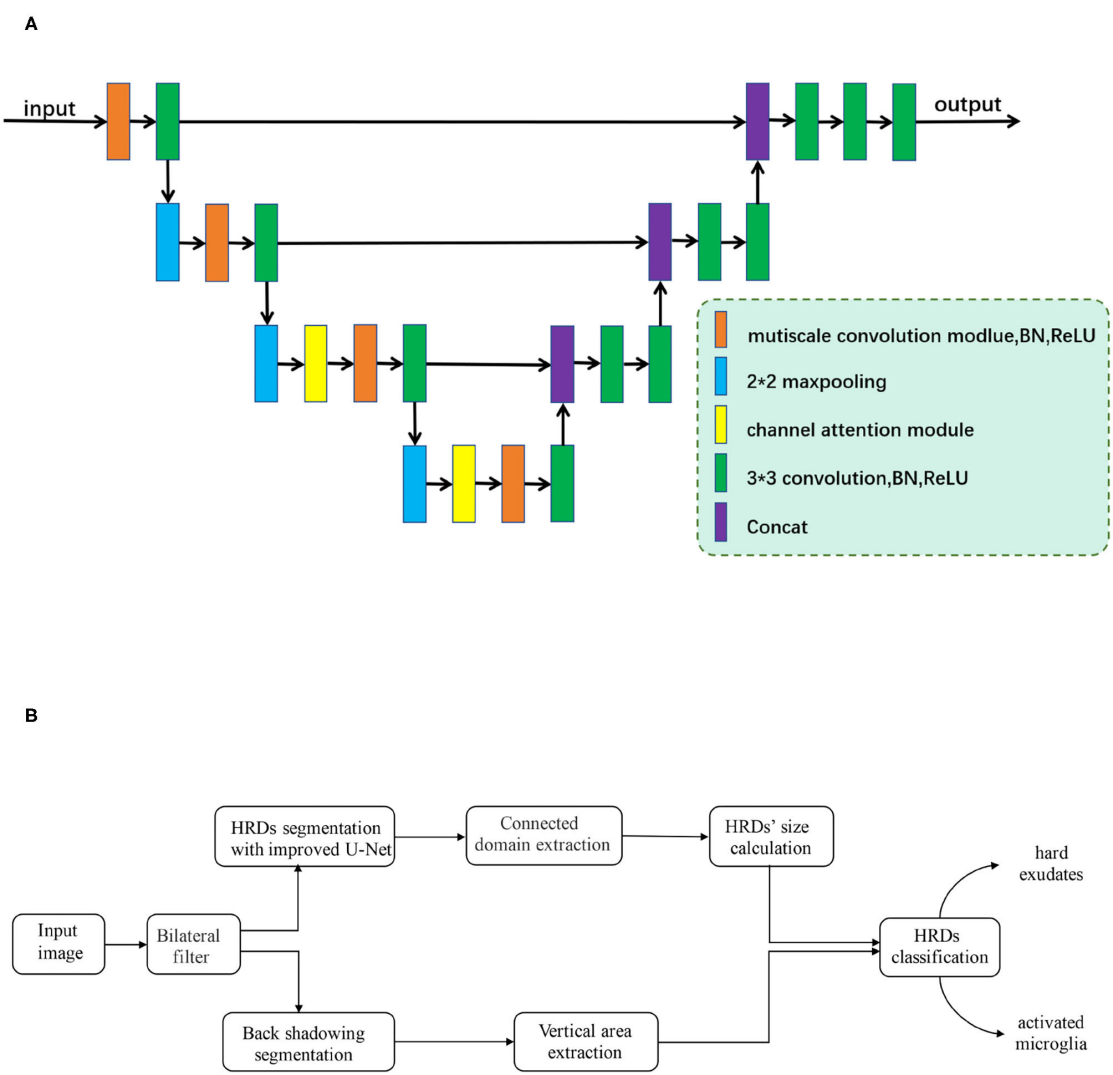
**(A)** The framework of the improved *U*-shaped convolutional neural network. Improved multi-scale convolution modules (MSCM), which are inserted into the encoder path of the network. Two channel attention modules (CAM), which are added to two max-pooling layers at the bottom. **(B)** Flowchart of proposed algorithm development.

In the first step, an improved *U*-shaped convolutional neural network (CNN), inspired by U-Net (25), was developed to segment HRDs on retinal OCT images, as is shown in Figure 2A. The original structure of U-Net consists of four downsampling and upsampling steps. Considering the size of HRDs, the number of downsampling and upsampling steps was reduced to three in our improved CNN. To deal with the variable size of HRDs, multi-scale convolution modules (MSCM) were proposed and inserted into the encoder path, which can help the network to achieve adaptive receptive fields. The MSCM is based on three parallel convolutions with dilation rates of 1, 2, and 3 so that multi-scale receptive fields can be obtained and network parameters can be reduced effectively. In addition to the MSCM, a channel attention module (CAM) was also designed to discard redundant information and guide the model to focus on the useful channel information. The CAM is constituted by a global average-pooling layer and a fully connected layer. It is an “end-to-end” structure and can be easily inserted in the CNN. Experiments show that CAM has a better effect on high-level semantic information, so two CAMs were inserted in the bottom two encoders. With these two innovative structures, automatic segmentation of HRDs was improved.

In the second step, a connected domain extraction algorithm was used to mark each HRD. Connected domain generally refers to the area on an image composed of adjacent pixels with the same pixel value. Connected domain extraction is the process of recognizing each connected area on an image. Based on different spatial adjacent morphological characteristics of hard exudates and small HRDs, an edge extraction network was developed, which contains edge guide branches to segment the back shadowing on retinal OCT images. For segmentation of small lesions, this network extracts the edge of the gold standard area marked by rater 1 as an auxiliary condition training model, which can improve the accuracy of the network segmentation of the lesion.

After segmentation of back shadowing, automatic classification of the HRDs segmentation results in the first step was achieved by the following strategy: (1) HRDs larger than 40 μm with back shadowing are classified as hard exudates. (2) HRDs smaller than 20 μm are regarded as noise and excluded. (3) HRDs which are neither hard exudates nor noise are recognized as small HRDs.

The whole experiment is computed under the environment of python and pytorch framework. The flowchart of the algorithm development is shown in Figure 2B.

This algorithm uses a 4-fold cross-validation method. The data set is equally divided into four parts and marked as f1, f2, f3, and f4. Three parts are used as training set to get a convergent model and the last part is used as test set to test the accuracy of the model. This method was repeated four times, each time using a different test set, i.e., f1, f2, f3, and f4, respectively. The average of the accuracy of each model was calculated as the final output. Compared with an independent training, validation, and test set, all data in cross-validation have undergone training and validation. If the overall data are unevenly distributed, cross-validation can produce an unbiased result and can be more universally used.

To improve the generalization of the network, we adopted online augmentation strategies including left and right flipping, up and down flipping, random rotation, and additive Gaussian noise addition. For each round of training, two to four of these augmentation methods are used. In the training process, the stochastic gradient descent (SGD) algorithm with an initial learning rate of 0.01, momentum of 0.9, and weight decay of 0.0001 is used to optimize the network. The batch size is set to 2 and the number of epochs is 60.

### Statistical Analysis

The sample size of this study was based on a previously published algorithm focusing on a similar topic (19). The output of proposed algorithm includes the area and number of total HRDs, hard exudates, and small HRDs, respectively, within 6, 3, and 1 mm diameters centered on the fovea. All the parameters were evaluated with histograms for distribution patterns. Mean and SD were applied to describe normally distributed data, while median and interquartile ranges (IQRs) were used to describe non-normally distributed data. Repeatability of the aforementioned parameters between two methods (either between two raters or between one rater and proposed algorithm) was evaluated with Dice coefficients and intraclass correlation coefficients (ICCs). A Dice coefficient is a metric used to evaluate the overlapping of the same target labeled by two methods. It ranges from 0 to 1. The higher the Dice coefficient, the better the two methods are overlapping. The correlation between Dice coefficient and area of total HRDs was evaluated by a correlation coefficient. Correlation and agreement of the parameters between two methods was analyzed with linear regression and Bland–Altman plots. SPSS 23.0 software was used for all statistical analyses. *P*-values <0.05 were considered statistically significant.

## Results

Twenty OCTs from 20 DME patients were included in this study (12 right and eight left eyes). If one patient had DME in both eyes, one eye was selected at random. Fifteen patients were treatment-naïve, four patients received macular laser, and one patient had anti-VEGF injections before inclusion. Eleven eyes were diagnosed with non-proliferative diabetic retinopathy and nine eyes had proliferative diabetic retinopathy. The mean central retinal thickness within 6 mm diameter of the ETDRS circle was 334 μm (IQR 314–390 μm) and the mean volume of the 6 mm ETDRS circle was 9.45 mm^3^ (IQR 8.88–11.04 mm^3^). The mean score of the Top Q Image Quality was 38 ± 5. The descriptive data of HRD parameters of rater 1, rater 2, and proposed algorithm of 6 × 6 mm area, within 1 mm and 3 mm diameters centered on the fovea, are presented in Table 1.

**Table 1:** Descriptive data of HRD parameters of rater 1, rater 2, and proposed algorithm of 6^*^6 mm area, within 1 and 3 mm diameters centered on fovea.

	**Rater 1**	**Rater 2**	**Proposed algorithm**
6 m^*^6 mm area centered on fovea			
Total HRD area (mm^2^)	0.61 (IQR: 0.43–1.45)	0.46 (IQR: 0.31–1.41)	0.62 (IQR: 0.33–1.46)
Total HRD number	468 (IQR: 294–800)	518 (IQR: 358–872)	554 (IQR: 306–795)
Hard exudate area (mm^2^)	0.35 (IQR: 0.08–0.83)	0.15 (IQR: 0.05–0.73)	0.38 (IQR: 0.13–0.89)
Hard exudate number	88 (IQR: 29–213)	45 (IQR: 17–200)	85 (IQR: 40–231)
Small HRD area (mm^2^)	0.32 (IQR: 0.22–0.53)	0.29 (IQR: 0.25–0.54)	0.29 (IQR: 0.18–0.45)
Small HRD number	368 (IQR: 258–606)	471 (IQR: 347–690)	440 (IQR: 250–646)
**Within 3 mm diameters centered on fovea**			
Total HRD area (mm^2^)	0.19 (IQR: 0.11–0.38)	0.15 (IQR: 0.08–0.37)	0.14 (IQR: 0.09–0.43)
Total HRD number	142 (IQR: 80–316)	179 (IQR: 97–275)	119 (IQR: 88–316)
Hard exudate area (mm^2^)	0.09 (IQR: 0.03–0.24)	0.11 (IQR: 0.04–0.29)	0.08 (IQR: 0.03–0.14)
Hard exudate number	33 (IQR: 9–114)	75 (IQR: 32–189)	23 (IQR: 11–84)
Small HRD area (mm^2^)	0.10 (IQR: 0.05–0.16)	0.04 (IQR: 0.01–0.06)	0.07 (IQR: 0.05–0.16)
Small HRD number	105 (IQR: 62–149)	50 (IQR: 24–110)	95 (IQR: 62–236)
**Within 1 mm diameters centered on fovea**			
Total HRD area (mm^2^)	0.010 (IQR: 0.004–0.048)	0.011 (IQR: 0.003–0.029)	0.011 (IQR: 0.003–0.033)
Total HRD number	11 (IQR: 5–34)	18 (IQR: 5–26)	12 (IQR: 4–41)
Hard exudate area (mm^2^)	0.015 (IQR: 0–0.097)	0.004 (IQR: 0.001–0.018)	0.003 (IQR: 0–0.017)
Hard exudate number	1 (IQR: 0–9)	5 (IQR: 1–16)	1 (IQR: 0–6)
Small HRD area (mm^2^)	0.006 (IQR: 0.003–0.018)	0.004 (IQR: 0.001–0.008)	0.004 (IQR: 0.001–0.017)
Small HRD number	9 (IQR: 4–23)	7 (IQR: 1–11)	8 (IQR: 3–28)

Table 2 shows the Dice coefficients and correlations of all parameters between two methods. The mean Dice coefficient of total HRD area between the two raters was 0.59 ± 0.14, while the Dice coefficients of hard exudate area and small HRD area were 0.58 ± 0.12 and 0.38 ± 0.10, respectively. The Dice coefficient between rater 1 and proposed algorithm of total HRD area was 0.70 ± 0.10, and the Dice coefficients of hard exudate area and small HRD area were 0.72 ± 0.11 and 0.46 ± 0.06, respectively. The Dice coefficient of total HRD area between rater 1 and proposed algorithm was correlated with total HRD area labeled by rater 1 (*r* = 0.48, *p* = 0.03) and by proposed algorithm (*r* = 0.52, *p* = 0.02).

**Table 2:** Reliability and correlation of HRD parameters of 6^*^6 mm area centered on fovea between two methods.

**Parameters**	**Total HRD area**	**Hard exudate area**	**Small HRD area**	**Total HRD number**	**Hard exudate number**	**Small HRD number**
**Dice coefficient (SD)**						
Between 2 raters	0.593 (0.136)	0.580 (0.116)	0.375 (0.102)	NA	NA	NA
Between rater 1 and algorithm	0.695 (0.103)	0.724 (0.106)	0.460 (0.056)	NA	NA	NA
**ICC (95% CI)**						
Between 2 raters	0.930 (0.822–0.972)	0.860 (0.546–0.950)	0.953 (0.881–0.981)	0.950 (0.873–0.980)	0.917 (0.642–0.973)	0.906 (0.764–0.963)
Between rater 1 and algorithm	0.997 (0.993–0.999)	0.997 (0.993–0.999)	0.972 (0.843–0.991)	0.986 (0.965–0.995)	0.996 (0.990–0.998)	0.977 (0.942–0.991)
Between rater 2 and algorithm	0.927 (0.816–0.971)	0.886 (0.712–0.955)	0.943 (0.857–0.978)	0.942 (0.853–0.977)	0.948 (0.870–0.980)	0.898 (0.742–0.960)
**Correlation coefficient** **(all ***p*****<** 0.001)**						
Between 2 raters	0.962	0.946	0.906	0.906	0.951	0.842
Between rater 1 and algorithm	0.995	0.996	0.971	0.973	0.992	0.953
Between rater 2 and algorithm	0.965	0.954	0.901	0.892	0.961	0.819

*HRD, hyperreflective dots; SD, standard deviation; NA, not applicable; ICC, intraclass correlation coefficient; CI, confidence interval*.

**Figure 3:**
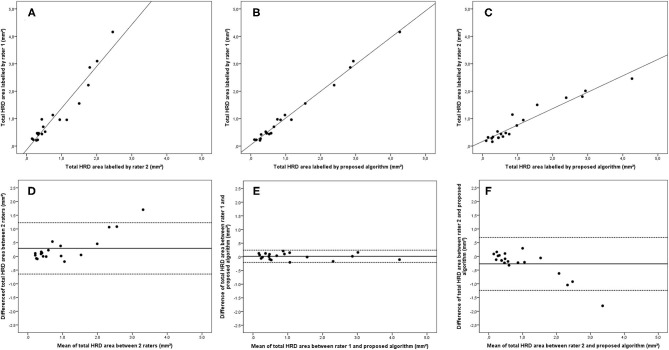
Linear regression and Bland–Altman analysis for correlation of total HRD area **(A)** between 2 raters, **(B)** between rater 1 and proposed algorithm, and **(C)** between rater 2 and proposed algorithm; agreement of total HRD area **(D)** between 2 raters, **(E)** between rater 1 and proposed algorithm, and **(F)** between rater 2 and proposed algorithm.

**Figure 4:**
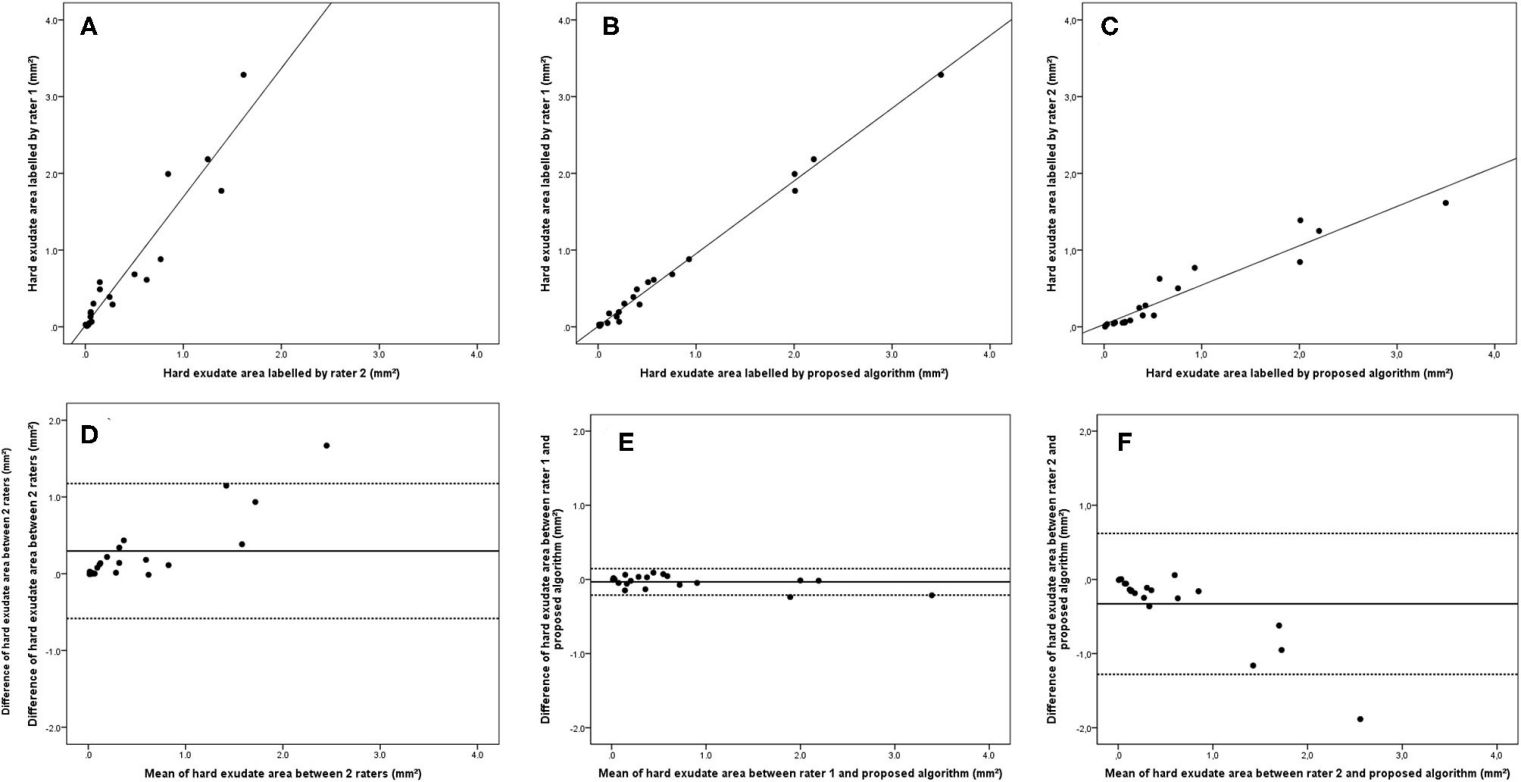
Linear regression and Bland–Altman analysis for correlation of hard exudate area **(A)** between 2 raters, **(B)** between rater 1 and proposed algorithm, and **(C)** between rater 2 and proposed algorithm; agreement of total HRD area **(D)** between 2 raters, **(E)** between rater 1 and proposed algorithm, and **(F)** between rater 2 and proposed algorithm.

**Figure 5:**
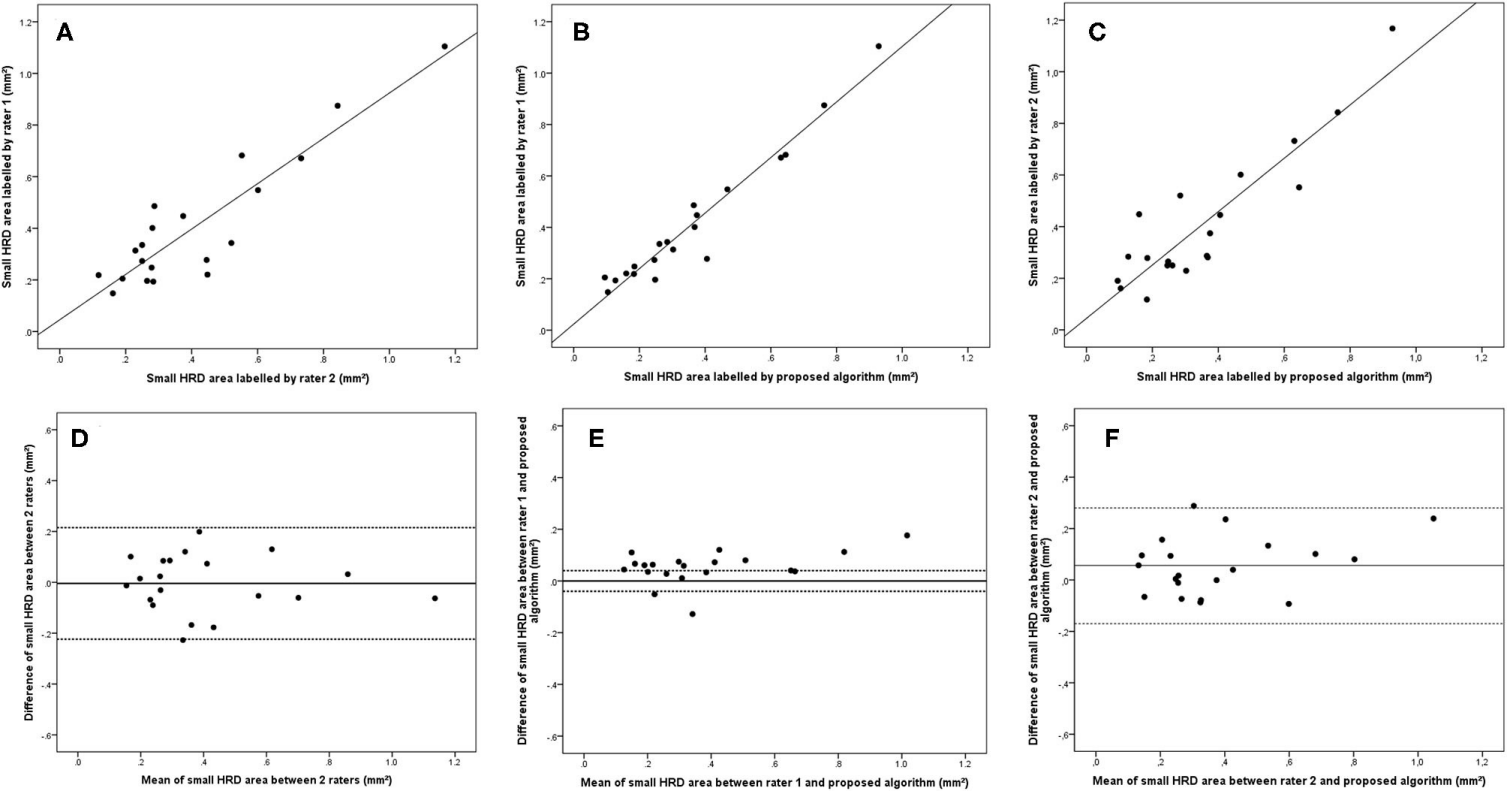
Linear regression and Bland–Altman analysis for correlation of small HRD area **(A)** between 2 raters, **(B)** between rater 1 and proposed algorithm, and **(C)** between rater 2 and proposed algorithm; agreement of total HRD area **(D)** between 2 raters, **(E)** between rater 1 and proposed algorithm, and **(F)** between rater 2 and proposed algorithm.

**Figure 6:**
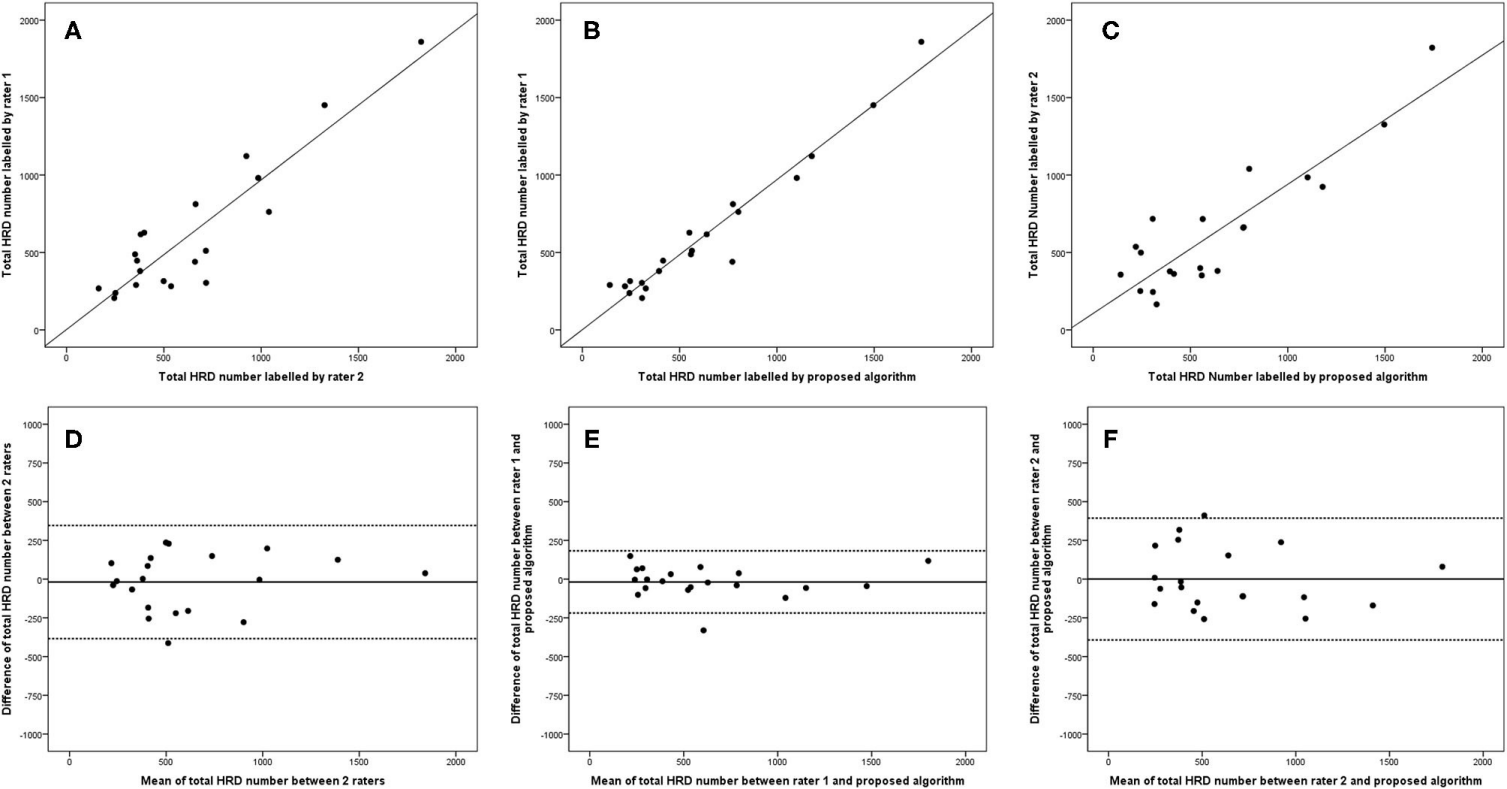
Linear regression and Bland–Altman analysis for correlation of total HRD number **(A)** between 2 raters, **(B)** between rater 1 and proposed algorithm, and **(C)** between rater 2 and proposed algorithm; agreement of total HRD area **(D)** between 2 raters, **(E)** between rater 1 and proposed algorithm, and **(F)** between rater 2 and proposed algorithm.

**Figure 7:**
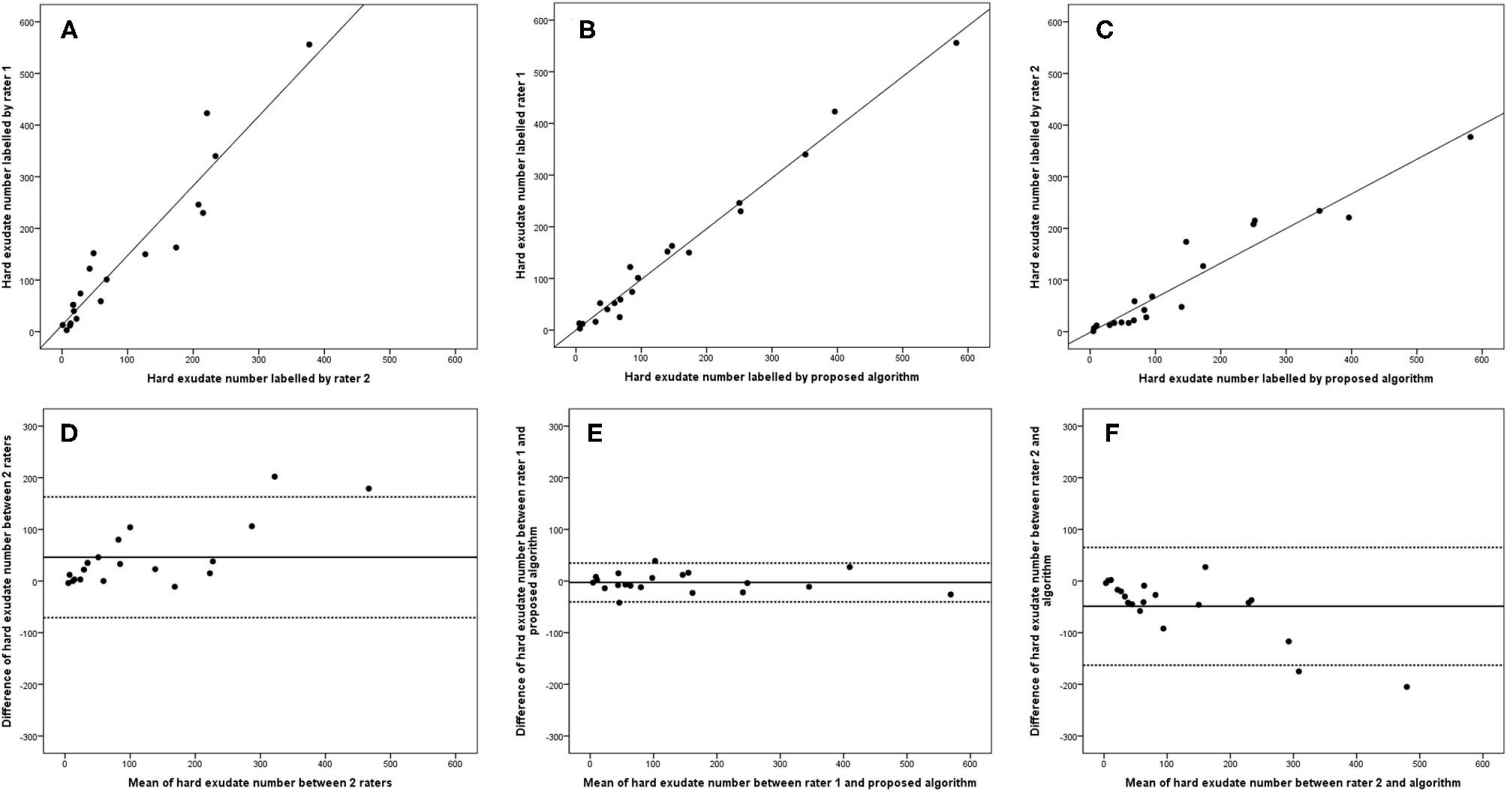
Linear regression and Bland–Altman analysis for correlation of hard exudate number **(A)** between 2 raters, **(B)** between rater 1 and proposed algorithm, and **(C)** between rater 2 and proposed algorithm; agreement of total HRD area **(D)** between 2 raters, **(E)** between rater 1 and proposed algorithm, **(F)** between rater 2 and proposed algorithm.

**Figure 8:**
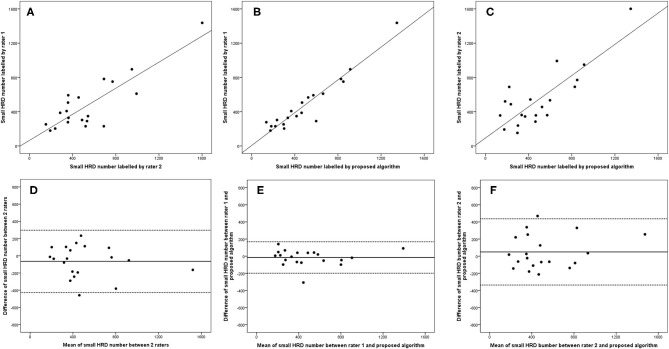
Linear regression and Bland–Altman analysis for correlation of small HRD number **(A)** between 2 raters, **(B)** between rater 1 and proposed algorithm, and **(C)** between rater 2 and proposed algorithm; agreement of total HRD area **(D)** between 2 raters, **(E)** between rater 1 and proposed algorithm, and **(F)** between rater 2 and proposed algorithm.

ICCs of all HRD parameters between the two raters ranged from 0.860 (95% CI: 0.546–0.950, hard exudate area) to 0.953 (95% CI: 0.881–0.981, small HRD area), and ICCs of the parameters between rater 1 and proposed algorithm ranged from 0.972 (95% CI: 0.843–0.991, hard exudate area) to 0.997 (95% CI: 0.993–0.999, small HRD area) (Figures 3–**5**). The correlations between rater 1 and proposed algorithm (range: 0.953–0.996, all *p* < 0.001) were stronger than the ones between the two raters (range: 0.842–0.962, all *p* < 0.001) for all parameters (Figures 3–**8**). As the algorithm was trained with data from rater 1, ICCs and correlations between rater 1 and the algorithm were stronger than those between rater 2 and the algorithm. However, ICCs and correlations between rater 2 and the algorithm were similar to those between the two raters.

Bland–Altman plots demonstrated that the 95% limits of agreement (LOA) of the total HRD area between rater 1 and proposed algorithm (range from −0.21 to 0.24) were much smaller compared with the 95% LOA of two measurements between the two raters (range from −0.64 to 1.23) (Figure 3). Similar differences were observed for the hard exudate area (Figure 4) and the small HRD area (Figure 5).

The 95% LOA of the total HRD number between rater 1 and proposed algorithm (range from −219 to 182) were also smaller compared with the 95% LOA of two measurements between the two raters (range from −384 to 346) (Figure 6). Similar differences are illustrated in Figure 7 for the numbers of hard exudates and in Figure 8 for the number of small HRDs.

The parameters within a 1 mm diameter and 3 mm diameter circle centered on the fovea also showed good reliability and correlation between rater 1 and proposed algorithm (Table 3). For the parameters measured within a 1 mm diameter circle centered on the fovea, ICCs of all HRD parameters between rater 1 and proposed algorithm ranged from 0.819 (small HRD area) to 0.994 (hard exudate area) (Table 3), while the correlation coefficient ranged from 0.694 (small HRD area) to 0.988 (hard exudate area). For the parameters measured within a 3 mm diameter circle centered on the fovea, ICCs of all HRD parameters between rater 1 and proposed algorithm ranged from 0.713 (small HRD area) to 0.989 (total HRD area) (Table 3), and the correlation coefficient ranged from 0.597 (small HRD area) to 0.979 (hard exudate area). The results within a 1 and 3 mm diameter area centered on the fovea were weaker than those within a 6 × 6 mm area, thus using the 6 × 6 mm area would be preferable for future clinical studies.

**Table 3:** Intraclass correlation coefficient of HRD parameters within 1 and 3 mm diameters centered on fovea between 2 raters and between rater 1 and proposed algorithm of parameters.

**ICC (95% CI)**	**Total HRD area**	**Hard exudate area**	**Small HRD area**	**Total HRD number**	**Hard exudate number**	**Small HRD number**
**Within 1 mm diameters centered on fovea**						
Between 2 raters	0.914 (0.783–0.966)	0.919 (0.796–0.968)	0.610 (0.014–0.846)	0.923 (0.807–0.970)	0.905 (0.761–0.963)	0.600 (−0.10–0.842)
Between rater 1 and algorithm	0.992 (0.981–0.997)	0.994 (0.985–0.998)	0.819 (0.543–0.928)	0.894 (0.733–0.958)	0.965 (0.912–0.986)	0.830 (0.571–0.933)
Between rater 2 and algorithm	0.915 (0.785–0.966)	0.927 (0.816–0.971)	0.470^*^ (−0.340–0.790)	0.893 (0.730–0.958)	0.861 (0.650–0.945)	0.425^*^ (−0.453–0.772)
**Within 3 mm diameters centered on fovea**						
Between 2 raters	0.913 (0.780–0.966)	0.931 (0.827–0.973)	0.347 (−0.649–0.742)	0.937 (0.840–0.975)	0.985 (0.961–0.994)	0.295 (−0.780–0.721)
Between rater 1 and algorithm	0.989 (0.971–0.995)	0.988 (0.970–0.995)	0.713 (0.274–0.886)	0.958 (0.895–0.983)	0.981 (0.952–0.993)	0.752 (0.374–0.902)
Between rater 2 and algorithm	0.921 (0.801–0.969)	0.949 (0.872–0.980)	−0.130^*^ (−1.854–0.553)	0.930 (0.824–0.972)	0.956 (0.890–0.983)	−0.081^*^ (−1.731–0.572)

* *P–value > 0.05; HRD, hyperreflective dots; ICC, intraclass correlation coefficient; CI, confidence interval*.

## Discussion

In the current study, a new algorithm for quantifying and differentiating HRDs on OCT in DME patients is introduced. All the parameters, including area and number of total HRDs, hard exudates, and small HRDs, show good correlation and agreement between the two raters and between the rater and proposed algorithm.

HRDs only visible on OCT in DME patients were first reported by Bolz in 2009 and hypothesized to be extravasated lipoproteins (7). However, more recent studies indicate that these structures represent microglia. Microglia are immunocompetent cells of retina (26) and are activated at different stages of human diabetic retinopathy (27). A recent study showed that these HRDs are strongly correlated with soluble CD 14 in aqueous humor, a cytokine released by microglia and macrophages, in DME patients (13). Moreover, these HRDs were also present in diabetic patients without manifest diabetic retinopathy and the number of HRDs increases as diabetic retinopathy progresses (28). Therefore, these HRDs on OCT are more likely to be activated microglia in DME patients.

For the proper development of an algorithm, the definitions of hard exudates and small HRDs must be rigorous and their OCT characteristics should not or only minimally overlap. We based our definition of hard exudates and small HRDs on previous studies. Definitions of HRDs representing activated microglia in the literature vary, and sizes ranging from 20 to 50 μm have been given (8, 10, 13, 24). However, a solid foundation of such definition is lacking. The reason could be that it is difficult to measure microglia in human retina by histology, especially the size of the entire microglial cells (cell body and dendrite ramifications). In contrast, it is possible to measure the cell bodies of the microglia (personal comment by M.O.M. Tso). The cell body of normal or quiescent microglia in human retina has a long axis of 16.4–20.4 μm and a short axis of 6–7.6 μm (29, 30). With inflammation such as DME, microglia will be activated and the size of cell body will enlarge, presenting as amoeboid morphology (26). By estimating the cell body of human activated microglia from histology pictures published by Zeng et al. (27), we defined small HRDs as structures of 20–40 μm length, which means a doubling of the size compared with quiescent microglial cells.

In previous studies, different algorithms for quantifying HRDs on OCT were developed. Our proposed algorithm showed similar Dice coefficients of total HRDs (0.695 ± 0.103) compared with published ones (0.638 and 0.713) (19, 20). The published algorithms did not differentiate HRDs into different types (18–20), which would provide ambient information for clinical analysis. Moreover, all the published algorithms quantified HRDs with conventional machine learning techniques, while our proposed algorithm uses deep learning technology, which provides further possibility for analysis using transfer learning on other OCT machines and in other retinal diseases, which can shorten the learning process compared with a new algorithm. For each new situation, i.e., for each different OCT machine and each retinal disease, the algorithm should be adapted based on a series of manually labeled OCT scans.

The present study found a better Dice coefficient of hard exudates (0.724 ± 0.106) compared with that of small HRDs (0.460 ± 0.056). Hard exudates showed larger signals (>40 μm) compared with small HRDs (20–40 μm), as defined earlier. We also reported a positive correlation between the Dice coefficient of total HRDs between rater 1 and the algorithm and total HRD area. Such a result is predictable as the smaller the labeled target, the more difficult to get a perfect output by the algorithm. Even if the Dice coefficient of small HRDs is not very high, the correlation and agreement between rater 1 and the algorithm are very good. No published algorithm has identified and quantified such a small target, so we could not evaluate it by comparison. Thus, we think the output of this algorithm is acceptable.

As shown in the Bland–Altman plot, the mean of the total number of HRDs is more consistent in all methods than the numbers of hard exudates and small HRDs separately. This is because it can be difficult to differentiate hard exudates from small HRDs by our definition. Some small hyperreflective signals presented unclear back shadowing, which contributes to different classifications of HRDs by raters, and probably by the algorithm. Another reason would be that some HRDs are too close to each other and were recognized as one rather than several by different raters and the algorithm.

We acknowledge several limitations, such as a relatively low dice coefficient of small HRDs and minor deviations of HRD number counting, as explained explicitly earlier. The distribution of HRDs on OCT in DME patients is currently unknown and could be variable among different populations, which may induce potential selection bias of the algorithm. We also would like to highlight that this study is the first to develop an algorithm to differentiate HRDs into hard exudates and small HRDs and quantify these signals in numbers and area on OCT in DME patients using deep learning technology. To summarize, the present study introduces a newly developed algorithm to quantify and differentiate HRDs on OCT for DME patients. Standardizing HRDs with automatic calculation will provide more objective and repeatable data for further investigation of DME and related diseases.

## Data Availability Statement

The raw data supporting the conclusions of this article will be made available by the authors, without undue reservation.

## Ethics Statement

The studies involving human participants were reviewed and approved by the Institutional Review Board of Joint Shantou International Eye Center (JSIEC) of Shantou University and the Chinese University of Hong Kong. Written informed consent for participation was not required for this study in accordance with the national legislation and the institutional requirements.

## Author Contributions

HH, LZ, XC, and HC contributed to conception and design of the study. HH, TL, LZ, and WZ organized the database. HH performed the statistical analysis and wrote the first draft of the manuscript. LZ and CY wrote sections of the manuscript. LL revised the manuscript. All authors contributed to manuscript revision, read, and approved the submitted version.

## Conflict of Interest

The authors declare that the research was conducted in the absence of any commercial or financial relationships that could be construed as a potential conflict of interest.

## Publisher's Note

All claims expressed in this article are solely those of the authors and do not necessarily represent those of their affiliated organizations, or those of the publisher, the editors and the reviewers. Any product that may be evaluated in this article, or claim that may be made by its manufacturer, is not guaranteed or endorsed by the publisher.
